# Next- and Third-Generation Sequencing Outperforms Culture-Based Methods in the Diagnosis of Ascitic Fluid Bacterial Infections of ICU Patients

**DOI:** 10.3390/cells10113226

**Published:** 2021-11-18

**Authors:** Hanna Goelz, Simon Wetzel, Negin Mehrbarzin, Stefan Utzolino, Georg Häcker, Mohamed Tarek Badr

**Affiliations:** 1 Institute of Medical Microbiology and Hygiene, Medical Center–University of Freiburg, Faculty of Medicine, University of Freiburg, 79104 Freiburg, Germany; hanna.goelz@uniklinik-freiburg.de (H.G.); simon.wetzel@web.de (S.W.); mehrbarzinnegin@googlemail.com (N.M.); georg.haecker@uniklinik-freiburg.de (G.H.); 2 Center of Surgery, Department of General and Visceral Surgery, Medical Center–University of Freiburg, Faculty of Medicine, University of Freiburg, 79104 Freiburg, Germany; stefan.utzolino@uniklinik-freiburg.de; 3 BIOSS Centre for Biological Signaling Studies, University of Freiburg, 79104 Freiburg, Germany; 4 IMM-PACT-Program, Faculty of Medicine, University of Freiburg, 79104 Freiburg, Germany

**Keywords:** ascitic fluid infections, intensive care unit, next-generation sequencing, nanopore, anaerobic bacteria, full length 16S rRNA sequencing, molecular diagnostics, metagenomics

## Abstract

Objectives: Infections of the ascitic fluid are serious conditions that require rapid diagnosis and treatment. Ascites is often accompanied by other critical pathologies such as gastrointestinal bleeding and bowel perforation, and infection increases the risk of mortality in intensive care patients. Owing to a relatively low success rate of conventional culture methods in identifying the responsible pathogens, new methods may be helpful to guide antimicrobial therapy and to refine empirical regimens. Here, we aim to assess outcomes and to identify responsible pathogens in ascitic fluid infections, in order to improve patients’ care and to guide empirical therapy. Methods: Between October 2019 and March 2021, we prospectively collected 50 ascitic fluid samples from ICU patients with suspected infection. Beside standard culture-based microbiology methods, excess fluid underwent DNA isolation and was analyzed by next- and third-generation sequencing (NGS) methods. Results: NGS-based methods had higher sensitivity in detecting additional pathogenic bacteria such as *E. faecalis* and *Klebsiella* in 33 out of 50 (66%) ascitic fluid samples compared with culture-based methods (26%). Anaerobic bacteria were especially identified by sequencing-based methods in 28 samples (56%), in comparison with only three samples in culture. Analysis of clinical data showed a correlation between sequencing results and various clinical parameters such as peritonitis and hospitalization outcomes. Conclusions: Our results show that, in ascitic fluid infections, NGS-based methods have a higher sensitivity for the identification of clinically relevant pathogens than standard microbiological culture diagnostics, especially in detecting hard-to-culture anaerobic bacteria. Patients with such infections may benefit from the use of NGS methods by the possibility of earlier and better targeted antimicrobial therapy, which has the potential to lower the high morbidity and mortality in critically ill patients with ascitic bacterial infection.

## 1. Introduction

Ascites is the abnormal accumulation of fluid in the abdomen. It is a common condition in cirrhotic liver disease [1] that may affect up to 50% of compensated liver disease patients [2]. Other possible causes include heart failure, tuberculosis, pancreatitis, cancer, and bowel perforation. Infection of the ascitic fluid is a serious complication associated with high morbidity and mortality [3]. Abdominal infections are among the most common infections in the intensive care unit (ICU) [4], and they carry a substantial increase in the risk of mortality [5,6].

Successful identification of pathogenic organisms in ascitic fluid infections is essential to guide antimicrobial therapy and to refine antibiotic treatment [7,8,9]. Precisely targeted treatment will have a positive impact on therapy outcome and reduce the emergence of resistant bacteria as well as the side effects of antibiotic therapy [10,11].

Standard microbiological culture-based diagnostic methods have limitations in the rapid identification of the causative pathogens in ascitic infections, as they are relatively slow (they typically take over 2 days) and culture positivity rates remain very low [12,13,14]. Therapy regimens, therefore, tend to be empiric in nature. A key factor is the low sensitivity of culture for many of the gut organisms, especially anaerobic bacteria, the main reservoir of bacterial translocations to the abdominal cavity and ascitic fluid [15].

Culture-independent approaches such as next-generation sequencing have enabled us to explore a wide range of bacteria that are difficult to grow in standard diagnostic culture [16], and they have illustrated the complex microbial communities in the ascitic fluid of patients, where they may contribute to infection outcome [17,18,19]. Despite their high sensitivity, these platforms require substantial time for the preparation and running of the test, as results can be only acquired at the end of the sequencing run, and they are mainly suitable for short read sequencing of specific regions of the 16S rDNA gene. This approach has been hypothesized to have lower power in inferring genus and species level taxonomic classification in comparison with the full-length gene [20,21]. The introduction of newer third-generation sequencing platforms such as Oxford Nanopore sequencing technology may help overcome these limitations. There, sequencing data can be analyzed in real-time, even with the additional benefit of the possibility of sequencing long-reads such as the full 16S rDNA gene [22]. These advantages underline the great potential of nanopore sequencing for outbreak surveillance [23,24], and the method has shown time and sensitivity advantages in other diseases [24,25,26].

Very few studies have compared the performance of short-read Illumina sequencing against long-read nanopore sequencing in the diagnosis of infections. In our study, we aimed to explore the association of the clinical characteristics of critically ill patients with ascitic fluid infections and evaluate the comparative performance of standard microbiology diagnostic culture methods with short-read Illumina and long-read nanopore sequencing methods.

## 2. Methods

### 2.1. Study Design and Ethics Statement

The study was carried out in the Medical Center of Freiburg University (the university hospital) surgical intensive care unit between October 2019 and March 2021. In patients who had undergone abdominal paracentesis for exclusion of secondary bacterial infections, excess ascitic fluid (at least 5 mL) was immediately frozen in −80 °C for metagenomic analysis. Samples had a median transport time of 3 h and 56 min. An overview of the study design can be seen in Supplementary Figure S1. The study was approved by the Ethics Committee, Medical Center—University of Freiburg, (registration number 246/19), and was registered at ClinicalTrials.gov (NCT04131751). Written informed consent was provided by all participants or their legal representatives, in accordance with the Declaration of Helsinki.

### 2.2. Clinical Information Acquisition

Clinical characteristics were extracted from the electronic health record. Medical charts and records were screened for antibiotic prescription and alcohol/nicotine consumption. We recorded white blood cell count (WBCC), C-reactive protein (CRP), and PCT (procalcitonin) levels in the timeframe of the ±7 days closest to the abdominal paracentesis.

### 2.3. Microbiological Culture-Based Methods and Microscopy

As part of standard care of microbiological diagnostics, ascitic fluid samples were examined microscopically (Gram staining, detection of granulocytes and bacteria) and plated on different cultural media such as Columbia blood (Thermo Scientific^TM^ Oxoid^TM^, Wesel, Germany), chocolate blood, MacConkey, and yeast extract cysteine blood agar plates (HCB; in-house), followed by incubation for at least 48 h under aerobic and anaerobic conditions. Inoculated brain heart infusion broth with 0.093% (*w*/*v*) agar was incubated for five days. Identification of growing microorganisms was obtained by matrix-assisted laser desorption ionisation-time-of-flight mass spectrometry (MALDI-TOF, Bruker Daltonics, Bremen, Germany).

### 2.4. Microbial Genomic DNA Preparation

Bacterial DNA was isolated using a QIAamp DNA Mini Kit (Qiagen GmbH, Hilden, Germany) using a modified protocol. In brief, the bacteria present in the ascitic fluid were pelleted by centrifugation for 10 min at 5000× *g*. Pellets were lysed using proteinase K and microbial cells were disrupted using bead beating BashingBead™ Lysis Tubes (Zymo Research, Irvine, CA, USA) on the Precellys Evolution homogenisator (Bertin Technologies, Montigny-le-Bretonneux, France) for four rounds of 1 min beating with 2 min breaks on ice. All isolation steps were controlled using a negative sample that contained only isolation buffer.

### 2.5. Bacterial Sequencing Using Short-Read 16S rDNA Sequencing

The bacterial hypervariable V1–V2 region was amplified from DNA templates (≤200 ng) using the primers 27F and 338R under the following conditions: 30 s at 98 °C; 30–40 cycles of 9 s at 98 °C, 30 s at 56 °C, and 30 s at 72 °C; final extension for 10 min at 72 °C, using the Phusion^®^ Hot Start II DNA High-Fidelity DNA Polymerase. In parallel to negative controls, a standard bacterial and fungal mock community (Zymo Research, Irvine, CA, USA) was used as a positive control in all PCRs and sequencing runs [27]. PCR products were enzymatically purified and barcodes containing Illumina sequencing adapters were added in a second PCR reaction using the Quick-16S NGS Library Prep Kit (Zymo Research, Irvine, CA, USA). PCR products were quantified on a 1.5% agarose gel and Qubit 4.0. fluorometer (Thermo Fisher Scientific, Waltham, MA, USA), then pooled to generate equimolar subpools. Where required, the final pooled library was extracted from agarose gel with the Qiagen MinElute Gel Extraction Kit (Qiagen GmbH, Hilden, Germany), then purified with Select-a-Size DNA Clean & Concentrator (Zymo Research, Irvine, CA, USA) according to the manufacturer’s protocol. Pooled libraries were quantified by a NEBNext library quantification kit (New England BioLabs GmbH, Frankfurt am Main, Germany) and analyzed on a QiaXcel advanced system (Qiagen GmbH, Hilden, Germany). The final library was sequenced using the MiSeq v2 reagent kit (500 cycles) (Illumina Inc., San Diego, CA, USA) on a MiSeq system with 10% PhiX spike-in.

### 2.6. Bacterial Sequencing Using Long-Read 16S rDNA Sequencing

The PCRs were conducted using the primer pair (27F and 1492R) spanning the whole 16S rRNA gene sequence. Sequences of the primers used in this study can be found in Supplementary Table S1. The library preparation kit (SQK-RAB204, Oxford Nanopore Technologies Inc., Oxford, UK) was used following the manufacturer’s protocols. Amplified fragments were checked on 1.5% agarose gels and PCR products (45 µL each) were purified using 30 µL of Agencourt AMPure XP beads (Beckman Coulter Inc., Beverly, MA, USA), and eluted in 10 μL of buffer solution (10 mM Tris-HCl pH 8.0, with 50 mM NaCl). Purified DNA was quantified using Qubit as above, and 5–50 fmol of pooled libraries were prepared for Oxford nanopore MinION sequencing by adding 1 µL of rapid adapter before sequencing. Prepared libraries were loaded on FLO-MIN106D R9.4 flow cells and sequenced for around 48 h or until not enough pores were available for sequencing.

### 2.7. Sequencing Analysis Pipeline

Raw fastq files’ read quality was assessed using FastQC [28] and MultiQC [29]. Illumina short-read raw data were trimmed from sequencing adapters using cutadapt [30]. Further quality control, trimming, and analysis of short-reads were done using the DADA2 analysis pipeline [31] and visualized using multiple packages in the R programming language on Linux environment [32,33]. Amplicon sequence variants (ASVs) were extracted from DADA2 and were assigned to taxonomy ranks using the Genome Taxonomy Database [34] and BLAST [35]. Long-read sequencing data acquisition and basecalling were performed using the Nanopore MinKNOW program, and initially assessed for quality using NanoPlot [36] and MultiQC. Taxonomy classification and quality control analysis of long-read sequences were performed using the BugSeq workflow [37,38].

### 2.8. Data Visualization and Statistics

Visualization and clustering of the samples were performed using heatmap methods implemented in the R packages pheatmap [39], ClustVis [40], and ggplot2 [41]. Statistics and graphs were made using GraphPad Prism V7 (GraphPad Software Inc., San Diego, CA, USA). Bars represent the mean and error bars show the standard error of the mean. Chi-squared and Fisher’s exact tests were used to assess the statistical difference between the groups for categorical variables. The Kruskal–Wallis test was used for significance testing of the differences between groups for continuous variables. All results were interpreted by two experienced clinical microbiologists for clinical relevance and identification of non-pathogenic skin flora or potential contaminants.

## 3. Results

### 3.1. Characteristics of the Study Cohort

**Figure 1:**
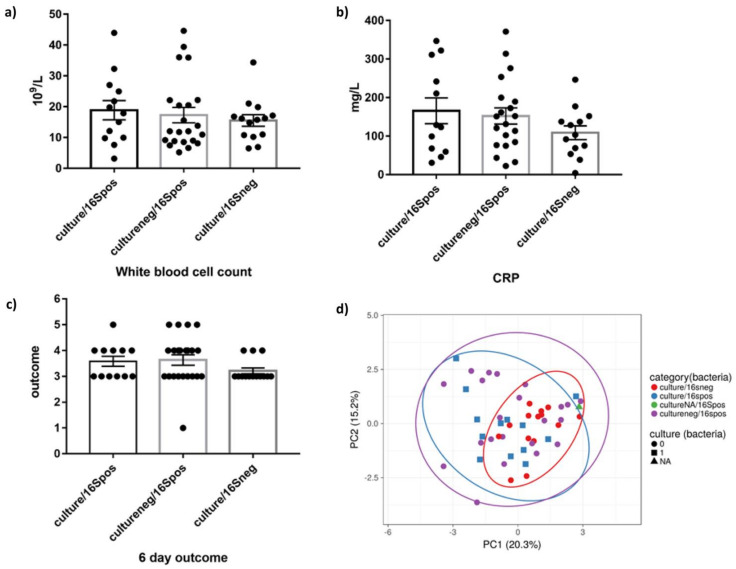
Comparison of clinical parameters between the study cohort groups. Patients were divided into three groups according to their microbiological culture and Illumina 16SrDNA PCR and sequencing results. (**a**–**c**) White blood cell count, CRP, and 6-day outcome. Data are presented as mean ± SEM. (**d**) PCA plot of study samples based on their clinical characteristics. The PCA plot shows first and second principal components, which explain 20.3% and 15.2% of the total variance, respectively.

After exclusion of non-eligible patients, a total of 50 patients were prospectively included in our study. To examine possible clinical correlations between patient characteristics and the identified organisms, patients were sub-grouped into three main categories: patients in whose sample both culture and sequencing yielded positive results, patients for whom only sequencing analysis detected bacteria, and patients whose samples were negative in both tests. The characteristics of the study cohort are shown in Table 1. Patients who were negative both in sequencing and culture had a lower white blood cell count, lower CRP, and overall a slightly better outcome, hinting at a possible active role for the microbes found by sequencing (Figure 1a–c). They also tended to have fewer granulocytes observed microscopically in their samples. Peritonitis and intestinal ischemia seem to be more common among the first two groups and not in the culture/16S rDNA negative group, indicating that sequencing could play an important role in the diagnosis and treatment of critically ill patients with secondary bacterial infections in these conditions, where only sequencing, but not cultural methods, identified potential pathogenic bacteria (7 of 50 patients in peritonitis). After grouping the patients based on their clinical characteristics using principle component analysis (PCA), all samples positive in sequencing clustered together, regardless of their culture status, but separate from sequencing negative samples (Figure 1d), suggesting a clinical correlation between the microbes found in sequencing and patients’ characteristics and outcome.

### 3.2. Culture of Ascites Samples

Of the 50 samples analyzed, 13 (26%) showed bacterial growth. *E. faecium*, *E. coli*, and *Klebsiella pneumonia* were among the most cultured bacteria. Only three samples showed growth of anaerobic bacteria, with *Lactobacillus* and *Clostridium clostridioforme.*

### 3.3. Generation of 16S rRNA Short and Long Read Sequencing Data

After DNA isolation and amplification, 36 of 50 (72%) samples had sufficient 16S rDNA amplicons to be suitable for sequencing together with positive and negative controls. Illumina 500 bp paired-end sequencing generated a total of 2,416,077 sequence reads and an average of 57,525 reads per sample. The 36 positive samples were also sequenced with nanopore 16Sr DNA long-read workflow, generating a total of 15,343,800 reads with an average of 426,216 and median of 52,500 reads per sample. The average quality of the sequenced samples can be seen in Supplementary Figure S2. All Illumina sequencing runs were controlled by negative and positive controls (mock community), where all bacterial members could be retrieved with a very good consensus with the predicted species distribution; Supplementary Figure S3.

### 3.4. Clinical Evalution of Short- and Long-Read Sequencing Output Compared with Standard Microbiology Culture Results

After filtering and merging of Illumina forward and reverse reads, reads found in negative controls were discarded from further analysis. Filtered reads were taxonomically assigned using the GTDB and BLAST databases. For short-read data, both GTDB and BLAST assignments were consolidated, and reads from similar species were merged. Species with less than 200 reads in all samples were ignored, as they are likely to be a contaminant. Taxonomic composition (phylum and family level) of the samples based on short-read sequencing can be seen in Supplementary Figures S4 and S5. The taxonomic composition (phylum and family level) of the long-read sequencing can be seen in Supplementary Figures S6 and S7. Identified bacteria were classified into one of four groups, either as primary pathogenic (commonly isolated in infectious diseases), anaerobic, normal-skin flora, or probably contaminant.

**Figure 2:**
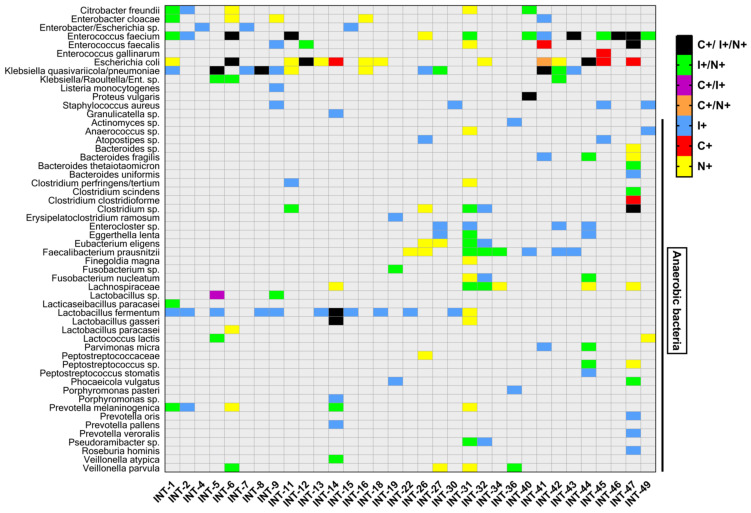
Pathogen identification through culture- and sequencing-based methods. The clinically most relevant pathogens and identification of anaerobic species in ascitic samples were evaluated according to their microbiological culture and Illumina short-read and nanopore long-read 16SrDNA PCR and sequencing results. The observed pathogens in ascitic samples are shown in the corresponding filled-in squares. C = culture-based identification, I = Illumina short-read sequencing, N = nanopore long-read sequencing, (+) = successful identification.

The top ten species in each sample identified with short-read sequencing were compared with the culture results and nanopore results for concordance of identified bacteria, and bacteria belonging to the first two groups (primary pathogenic or anaerobic) are shown in Figure 2. Detailed results of identified species in culture and sequencing can be found in Supplementary File S1.

### 3.5. Anaerobic Bacteria Identification

**Figure 3:**
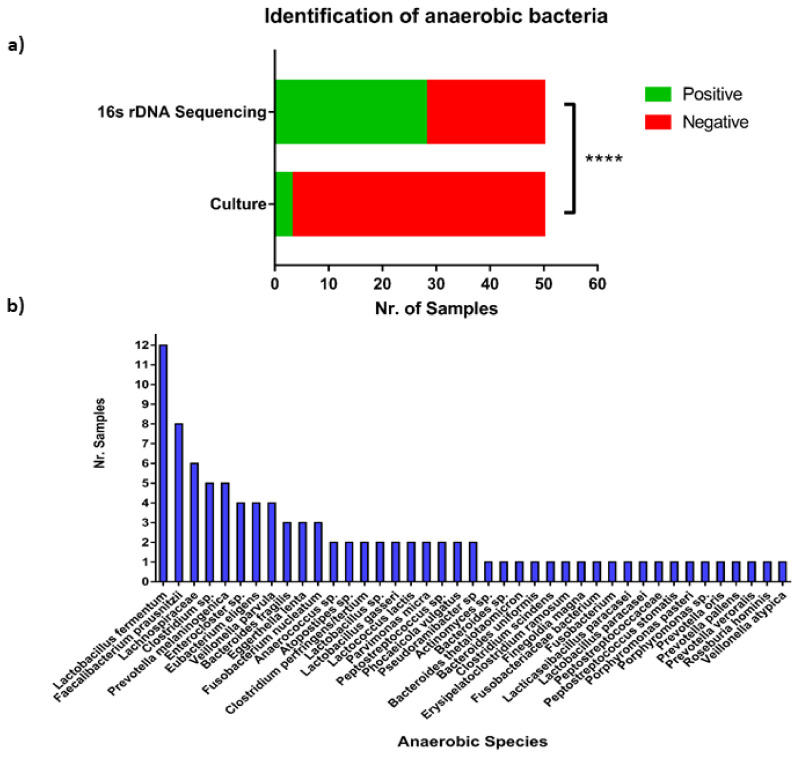
Frequency of anaerobic bacteria identification using culture- and sequencing-based methods. Patients were divided into three groups according to their microbiological culture and Illumina 16SrDNA PCR and sequencing results. (**a**) Frequency of patient samples where anaerobic bacteria could be identified using either culture-based or short-read sequencing methods. Significance was tested between the two groups using Fisher’s exact test (****, *p* < 0.0001). (**b**) The most common anaerobic bacteria identified by short-read sequencing in patient samples.

Besides conventional ascitic fluid pathogens, we could see a high number of species of anaerobic bacteria identified only through sequencing. In comparison with three samples showing cultural growth of anaerobic bacteria (6%), sequencing-based methods identified anaerobic bacteria in 28 samples (56%) (Figure 3a). Among those, *Lactobacilli* and *Faecalibacterium* were the most common genera (Figure 3b). When comparing the frequency of anaerobic bacteria identification, we see a very significant increase in their identification using NGS, suggesting a more prominent role for anaerobes in ascites pathogenesis than commonly appreciated, and a major restriction in the current standard care of microbiological diagnostics.

## 4. Discussion

Bacterial infection of ascitic fluid is a serious complication that is linked to poor clinical outcome and a significant increase in mortality, especially among patients in critical care units [4,42]. Microbiological diagnostics and identification of the causative bacteria can help improve patient outcome [43]. Our study shows the added advantage of applying next- and third-generation sequencing to the diagnosis of ascitic infections, especially among ICU patients. Various bacterial species that are normally overlooked were identified in the ascitic fluid, suggesting a potential pathogenic role for these bacteria.

A very important aspect of our sequencing workflow was the application of various positive and negative controls, in order to make the pipeline compatible for future integration into standard diagnostics. We thus used negative and positive controls to ensure the sensitivity of the amplification protocols while detecting possible contaminations. Such laboratory contaminations have been problematic in other cases, especially in low biomass samples such as clinical samples from primary sterile locations [44,45]. Indeed, we could identify and exclude from our analysis various common bacterial contaminants of sequencing workflows such as *Pseudomonadales*, *Deinococcales*, *Burkholderiales*, *Rhizobiales*, and *Sphingomonadales*, which have also been described as contaminants in previous studies [46,47].

The overall cultural positivity rate of our cohort was 26%, which is equivalent to rates reported previously [48]. We could amplify and identify bacteria in 36 (72%) samples. In most cases where both methods were positive, agreement between sequencing results and culturally grown bacterial was very good. It was surprising to us how many bacteria that can typically quite easily be cultured, such as *Enterobacterales*, were only recovered by sequencing. This may be because of prior antibiotic treatment of these patients. Interestingly, in the case of samples (INT-7) and (INT-40), where the patients had sepsis with *Klebsiella pneumonia* and *Citrobacter freundii*, respectively, they could not be detected in the ascitic fluid culture. However, both pathogens were identified in these patients’ ascitic samples by sequencing, suggesting that the ascitic infection was indeed the source of this patient’s septicemia and illustrating the added advantage of sequencing in these cases.

In two patients where enterococci were culturally grown, sequencing methods could detect them, but assigned them to different enterococci species, indicating a possible mis-assignment by MALDI or by sequencing. The main gap of sequencing methods was the detection of *E. coli*, where in three cases, it was only detected by culture. In those cases, sequencing detected many other species that did not grow in culture, indicating a possible overgrowth by *E. coli* in culture at the expense of other bacteria. When comparing Illumina short-read with nanopore long-read results, the quality of the nanopore-sequenced reads was lower in comparison with Illumina, yet the longer read length enabled a comparable bacterial identification, making nanopore sequencing a good method for clinical applications, especially with its shorter turnaround time; that is, sufficient sequencing reads could be obtained after 3–4 h, in comparison with Illumina workflow that always needed the full run period of 28 h before analysis could start [23]. In many cases, both methods agreed on the identified bacteria, even up to the species levels, which is essential in deciding on the suitable empirical treatment. Main differences were detected in some species such as *L. fermentum*, which was more often found by short-read sequencing, or in *E. coli*, which was more frequently detected in long-read sequencing. These discrepancies could be potentially inferred by the sensitivity bias of short-read sequencing of a specific hypervariable region towards different bacterial species, in comparison with the other hypervariable regions that may be used in microbiome studies [21,49], which makes long-read sequencing of the whole gene a more unbiased approach.

Although both culture- and sequencing-based methods could identify many organisms that are commonly associated with bacterial peritonitis and ascitic infections such as *E. coli*, *Klebsiella*, and *Enterococcus* spp., the main advantage of sequencing was in the surprising identification of many anaerobic bacteria in our samples through sequencing (56%) that were not identified by the culture methods (only 6% were found by culture). This suggests that the current view of common pathogens in peritonitis is artificially narrowed by the exclusive use of sequencing methods. The low culture-isolation rate in our study is consistent with the literature reports (2–3% of culture positive samples yield anaerobic bacteria) [50]. Low sensitivity thus appears to be a method-intrinsic problem. In terms of antibiotic therapy, however, this gap seems relevant, and anaerobic bacteria should be suspected even when not cultured from ascitic fluid.

*Lactobacilli*, *Colistridium*, *Prevotella,* and *Faecalibacterium prausnitzii* were among the most commonly detected anaerobic species in the sequencing data. *Faecalibacterium* is considered a standard member of the normal gut flora, with various protective associations against many diseases, but their intestinal increase has been associated with psoriasis [51], indicating possible immunomodulation capabilities. *Prevotella* is one of the dominant genera in the human gut, putting it in a position to influence many aspects of human health and disease [52]. Beside its role in various infectious processes, *Prevotella* has been found among other anaerobes such as *Fusobacterium nucleatum* to be associated with colon cancer [53]; its presence in the ascites may be linked to malignant disease.

While considering these results inferred by sequencing and taking our local antimicrobial susceptibility patterns of anaerobic bacteria into account [54], we see that ampicillin-sulbactam or piperacillin-tazobactam and metronidazol are very effective in covering most anaerobic bacteria. When taking the more common ascites pathogens into account such as *E. coli* or enterococci, ampicillin-sulbactam or piperacillin-tazobactam seem to be a very good empirical start point until targeted therapy can be started.

In addition to the potentially pathological bacteria, NGS detected various skin commensals and potential skin and environmental bacteria owing to its high sensitivity, which most likely would not normally play a major role in patient outcome or influence the antimicrobial therapy. Such identification poses a challenge in the differentiation of pathogens, physiological skin flora, and contaminants, as well as in the clinical interpretation of the results.

In comparison with cultural methods, it is expected that the limitations of NGS-based methods may be time and cost. In our study, library preparation, sequencing, and data analysis took approximately 4–5 days, but it is likely that this time could be reduced to 3 days in a well-established standard diagnostic workflow. Compared with Illumina short-read sequencing, nanopore long-read sequencing is even less time consuming thanks to its much shorter sequencing time; however, this comes at the expense of some read quality. Although short- and long-read sequencing has become less expensive in recent years, the cost per sample is still highly dependent on the number of samples and consumables used in the sequencing workflow. In addition, new skills are needed to perform the sequencing workflows and to interpret the sequencing results in a clinically relevant manner. Careful consideration of the analysis pipeline, as well as application of suitable positive and negative controls, are highly critical to ensure the integrity of the workflow and quality of results. Nevertheless, NGS-based methods, which we have shown to outperform standard cultural methods in the detection of pathogenic bacteria in ascites fluid infections, are very promising for future integration into standard microbiology laboratories.

## 5. Conclusions

The rapid optimization of empirical antimicrobial therapy is one of the main cornerstones of hospital antibiotic stewardship, which leads to better patient prognosis, less mortality, and avoidance of the selection and spread of resistant organisms. Ascitic infections pose a challenge to this concept as culture positivity rates are relatively low (~25–40% of cases) and appear to miss most anaerobic bacteria, which impedes the successful targeting of the specific responsible pathogens. We could show that sequencing-based methods outperform standard microbiological methods in identifying organisms that are likely causative of ascitic fluid infections, particularly in detecting anaerobic bacteria, suggesting that their actual role in the pathophysiology of ascites infections is underestimated. To the best of our knowledge, this is the first study to compare targeted bacterial short- and long-read sequencing of ascites samples in intensive care unit patients.

## Figures and Tables

**Table 1:** Characteristics of the study cohort.

	Culture/16s-pos	Culture-neg/16s-pos	Culture/16s-neg	*p*-Value
N	13	22	14	
Age (years)	63 (52.5–73)	72 (53.75–79)	62 (55.5–71)	0.45
Sex (male)	10 (77%)	12 (55%)	11 (79%)	0.23
Leucocytes (Tsd)	17.84 (9.85–25.98)	12.03 (8.733–22.1)	15.95 (10.63–17.79)	0.74
CRP	126 (61.65–293.9)	141.1 (78.4–197.2)	103.3 (61.15–144.8)	0.47
PCT	1.02 (0.715–1.715)	2.575 (0.415–7.983)	1.35 (0.3875–4.323)	0.56
Alcoholism	1 (8%)	6 (32%)	2 (17%)	0.27
Smoking	3 (23%)	9 (45%)	2 (18%)	0.22
Granulocytes (microscopic)	3 (1.5–3)	2.5 (1–3)	1.5 (1–2.25)	0.22
Hospital stay after paracenthesis (d)	27.5 (10.5–35)	14.5 (10.75–29.5)	12.5 (8.75–28)	0.48
ICU stay after paracenthesis (d)	4 (1.5–8.5)	4 (1.75–12)	2 (0.75–5.75)	0.33
6-day evaluation	3.5 (3–4)	4 (3–4)	3 (3–3.25)	0.31
ICU discharge (alive)	10 (77%)	17 (77%)	14 (100%)	0.15
Intestinal ischemia	2 (15%)	6 (27%)	0 (0%)	0.1
Tumor	6 (46%)	13 (59%)	9 (64%)	0.62
Peritonitis	8 (62%)	7 (32%)	1 (7%)	0.01
Cirrhosis	1 (8%)	1 (5%)	2 (14%)	0.58
Antibiotictherapy (+5 d)	11 (92%)	12 (63%)	9 (64%)	0.19
Blood culture positivity (±5 d)	4 (40%)	5 (29%)	1 (13%)	0.44

Continuous data are reported as medians and interquartile ranges (IQRs), and significance was tested with Kruskal–Wallis test. Categorical data are presented as frequency and percentages, and was significance tested with chi-squared test. d = days; Tsd = thousand. Granulocytes amount was evaluated by gram stain microscopy (100x) according to the following scheme: 0 = no granulocytes; 1 = 1–24 cells; 2 = 25–99 cells; and 3 = ≥100 cells. Patient outcome was evaluated six days after paracentesis on a scale of 1–5, where one indicates patient release from hospital, two indicates discharge to a non-tertiary care hospital, three indicates release from intensive care to a normal hospital ward, four indicates continued need for intensive care, and five indicates that the patient was deceased. Blood culture positivity was evaluated for blood samples withdrawn in a five-day window around paracentesis in patients where sepsis was suspected.

## Data Availability

The raw sequences generated in this project were deposited in the European Nucleotide Archives (ENA) and available under study number PRJEB47278 and samples are available through accession numbers ERS7300144–ERS7300188. The code for this analysis is available on GitHub and can be accessed using the following link: https://github.com/Tarek-Badr/microbial-community-analysis-Pipeline (accessed on 26 October 2021).

## Supplementary Materials

The following are available online at https://www.mdpi.com/article/10.3390/cells10113226/s1, File S1: Detailed results of identified species in culture and sequencing methods of ascitic samples and parallel blood sample cultures, Table S1: Summary of primers used in the study, Figure S1: Study design, Figure S2: QC of Illumina and nanopore sequencing data, Figure S3: Mock community analysis using Illumina V1-2 16S rDNA sequencing, Figure S4: Taxonomic composition of short-read sequencing data at the phylum level, Figure S5: Taxonomic composition of short-read sequencing data at the family level, Figure S6: Taxonomic composition of long-read sequencing data at the phylum level, Figure S7: Taxonomic composition of long-read sequencing data at the family level.
