# Supplementary material for: Next- and Third-Generation Sequencing Outperforms Culture-Based Methods in the Diagnosis of Ascitic Fluid Bacterial Infections of ICU Patients
Source: Cells. 2021 Nov 18;10(11):3226. doi: 10.3390/cells10113226 (PMC8617993; doi:10.3390/cells10113226)
Supplement: Supplementary file 1 [file cells-10-03226-s001.zip › Supplements.pdf]

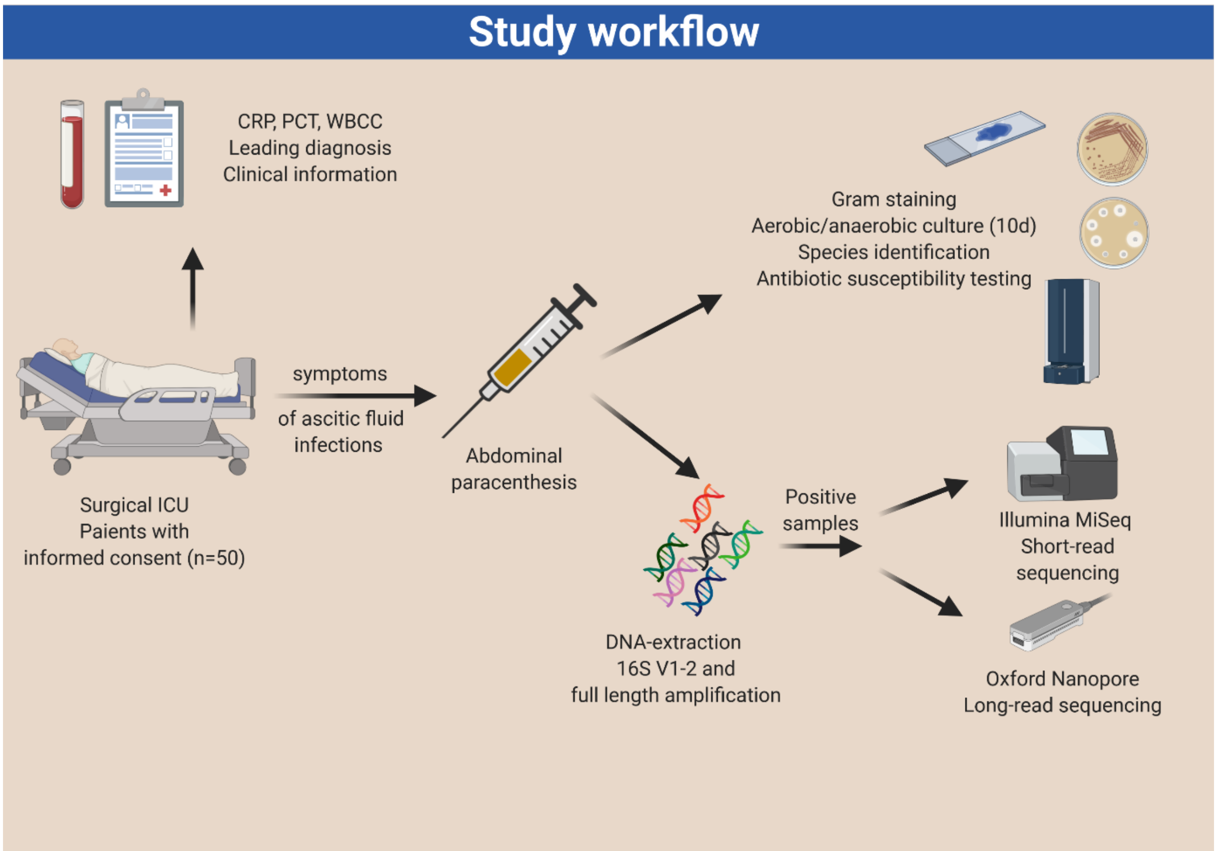

Supplementary Figure S1. Study design

Patients who underwent abdominal paracentesis in the Medical Center of Freiburg University surgical intensive care unit were screened for inclusion eligibility. For patients deemed eligible, informed consent was obtained and excess abdominal fluid was used for sequencing in addition to the standard of care culture-based microbiological diagnostics. DNA extraction was carried and 16s rDNA was amplified using V1-2 primers for short-read Illumina sequencing, or primers spanning the whole 16s rDNA gene for long-read Nanopore sequencing. Clinical parameters were extracted from the electronic health records such as the leading diagnosis, white blood cell count (WBCC), C-Reactive Protein (CRP), and PCT (Procalcitonin). “Created with BioRender.com.”

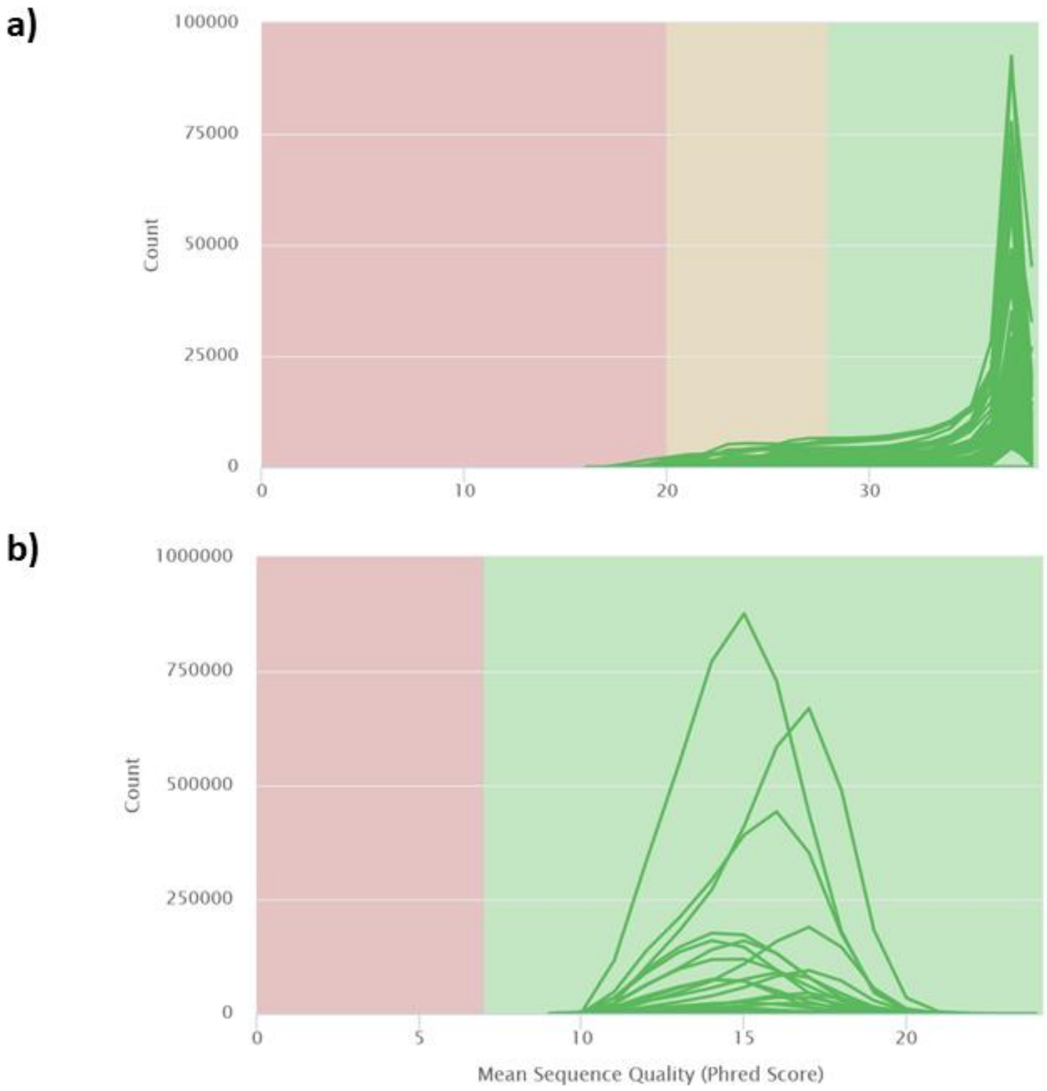

Supplementary Figure S2. Quality control of a) Illumina sequencing reads and b) Nanopore sequencing reads.

Quality control was done with multiQC and FastQC tools. The x-axis shows the per sequence quality score (phred score) and the y-axis shows the number of reads. Further analysis was carried out with reads successfully passing the quality control (green zone).

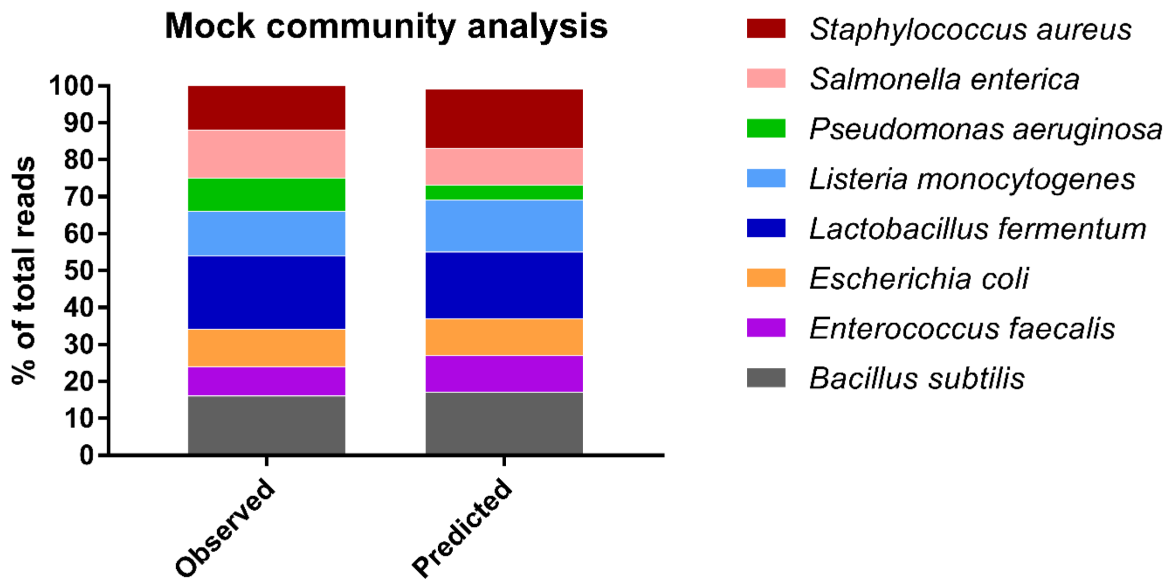

Supplementary Figure S3. Mock community analysis using Illumina V1-2 16S rDNA sequencing.

Analysis of average identification of different bacterial species in Zymo Mock community used as a positive control in all Illumina sequencing runs, and its comparison with the original microbial distribution.

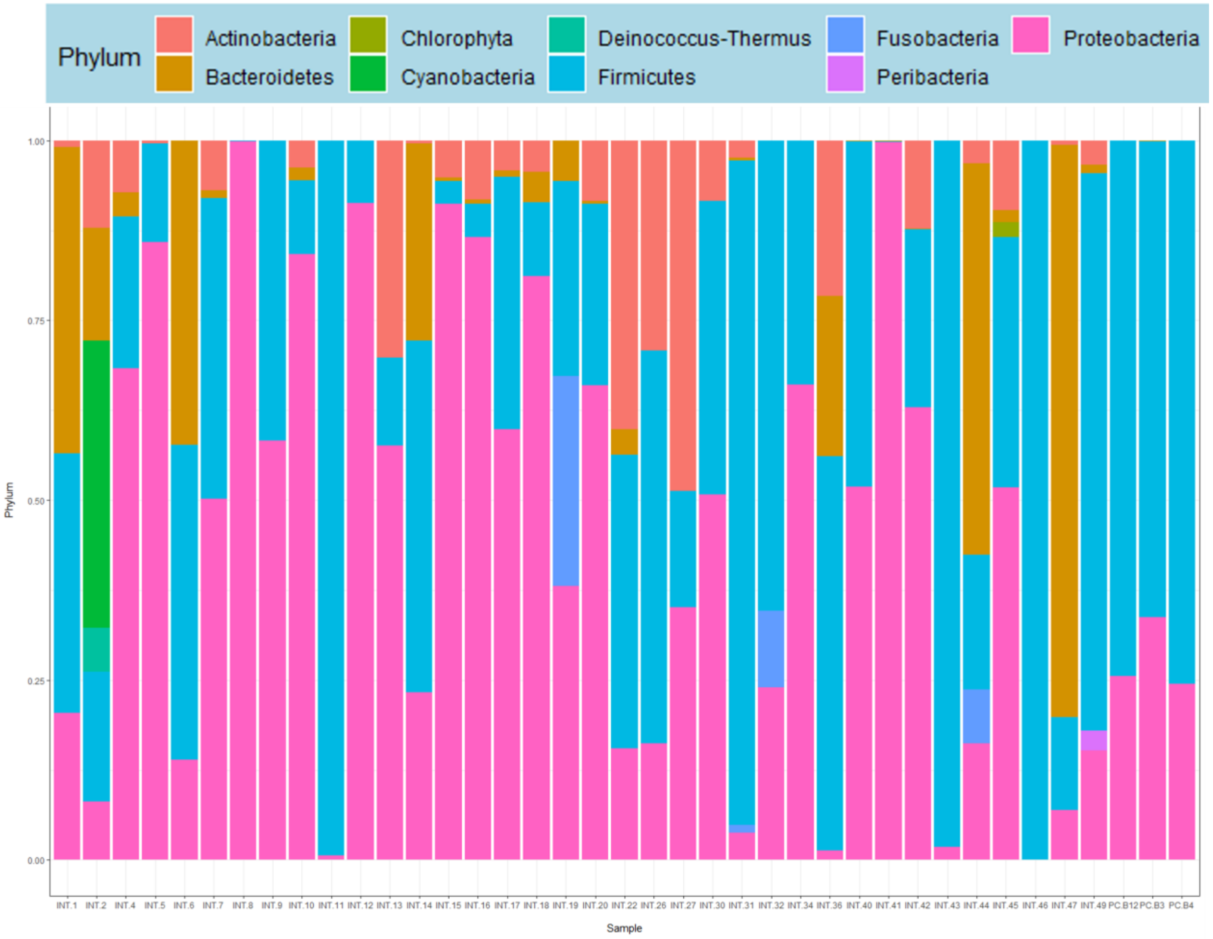

Supplementary Figure S4. Taxonomic composition of short-read sequencing data at the Phylum level

Bar plot showing the relative proportions of the phyla within all the samples. ASVs count and classification were constructed using DADA2 pipeline, and ASVs apparent in negative controls, or have lower than 200 reads in all samples were filtered out. PC = Zymo Mock community positive control. INT = ascites sample.

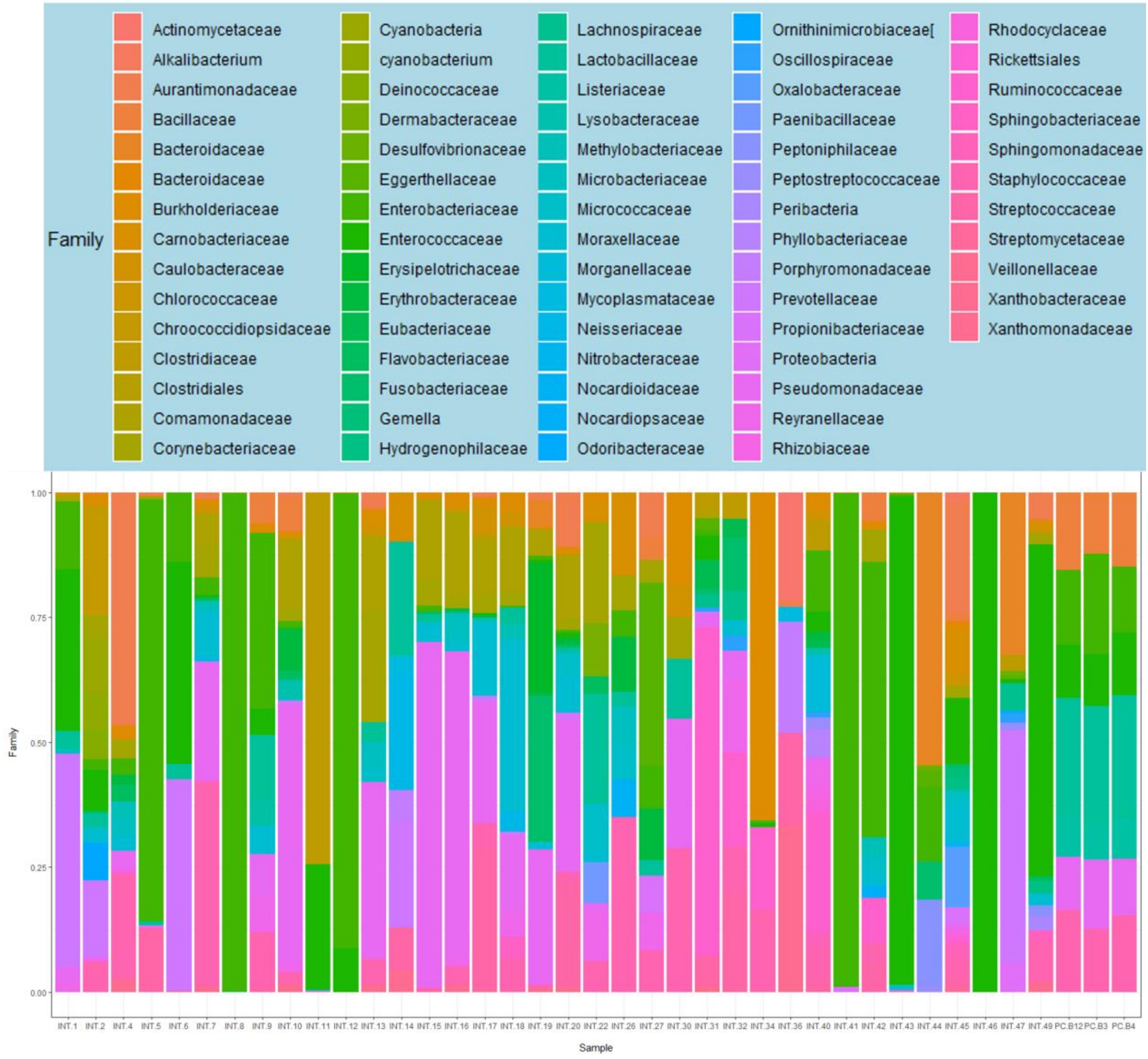

Supplementary Figure S5. Taxonomic composition of short-read sequencing data at the Family level

Bar plot showing the relative proportions of the families within all the samples. ASVs count and classification were constructed using DADA2 pipeline, and ASVs apparent in negative controls, or have lower than 200 reads in all samples were filtered out. PC = Zymo Mock community positive control. INT = ascites sample.

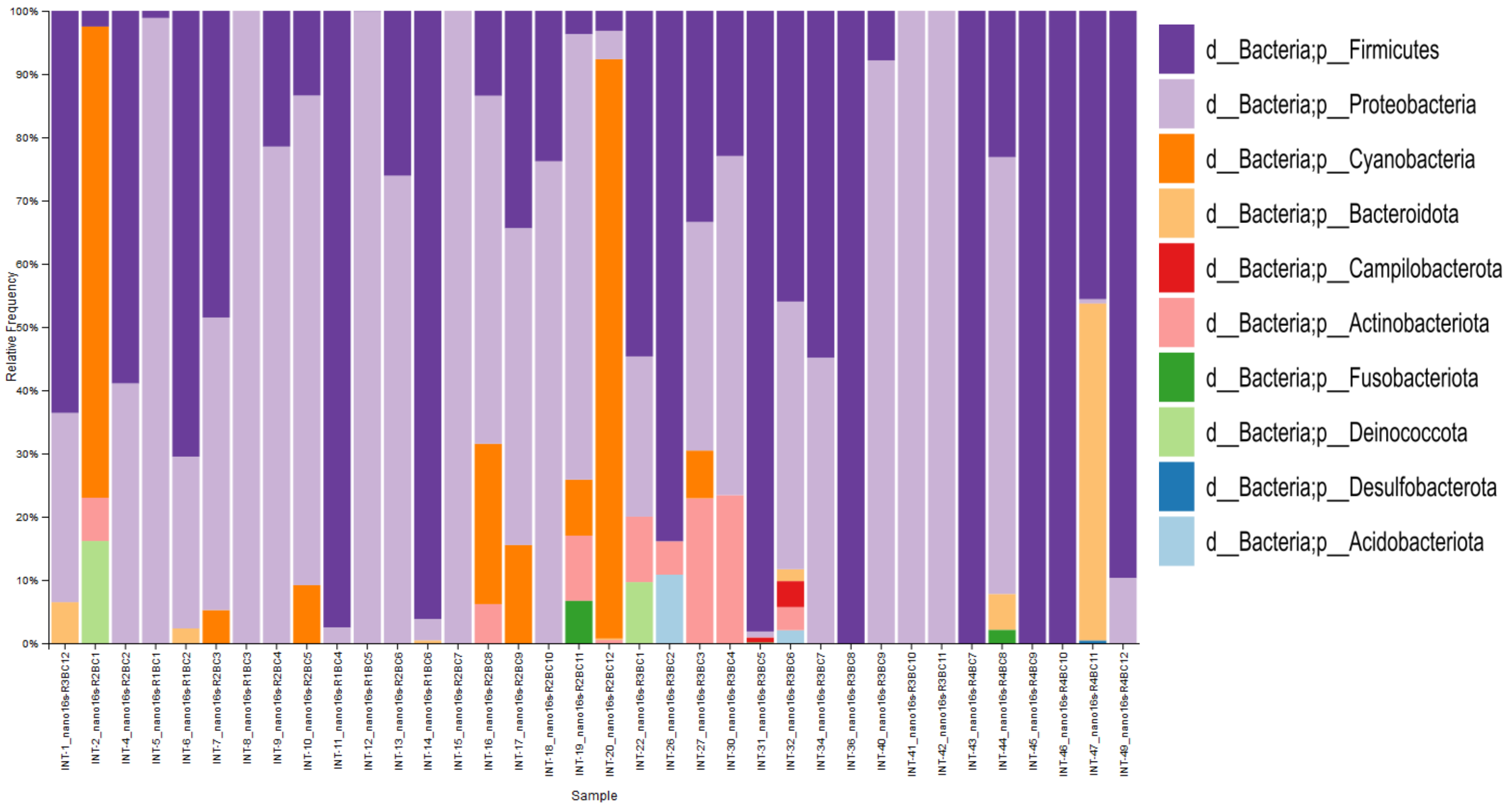

Supplementary Figure S6. Taxonomic composition of long-read sequencing data at the Phylum level

Bar plot showing the relative proportions of the phyla within all the samples based on 16S rDNA Nanopore sequencing. Initial reads demultiplexing and analysis was done on Nanopore Minknow, and taxonomic classification was carried out using the Bugseq pipeline.

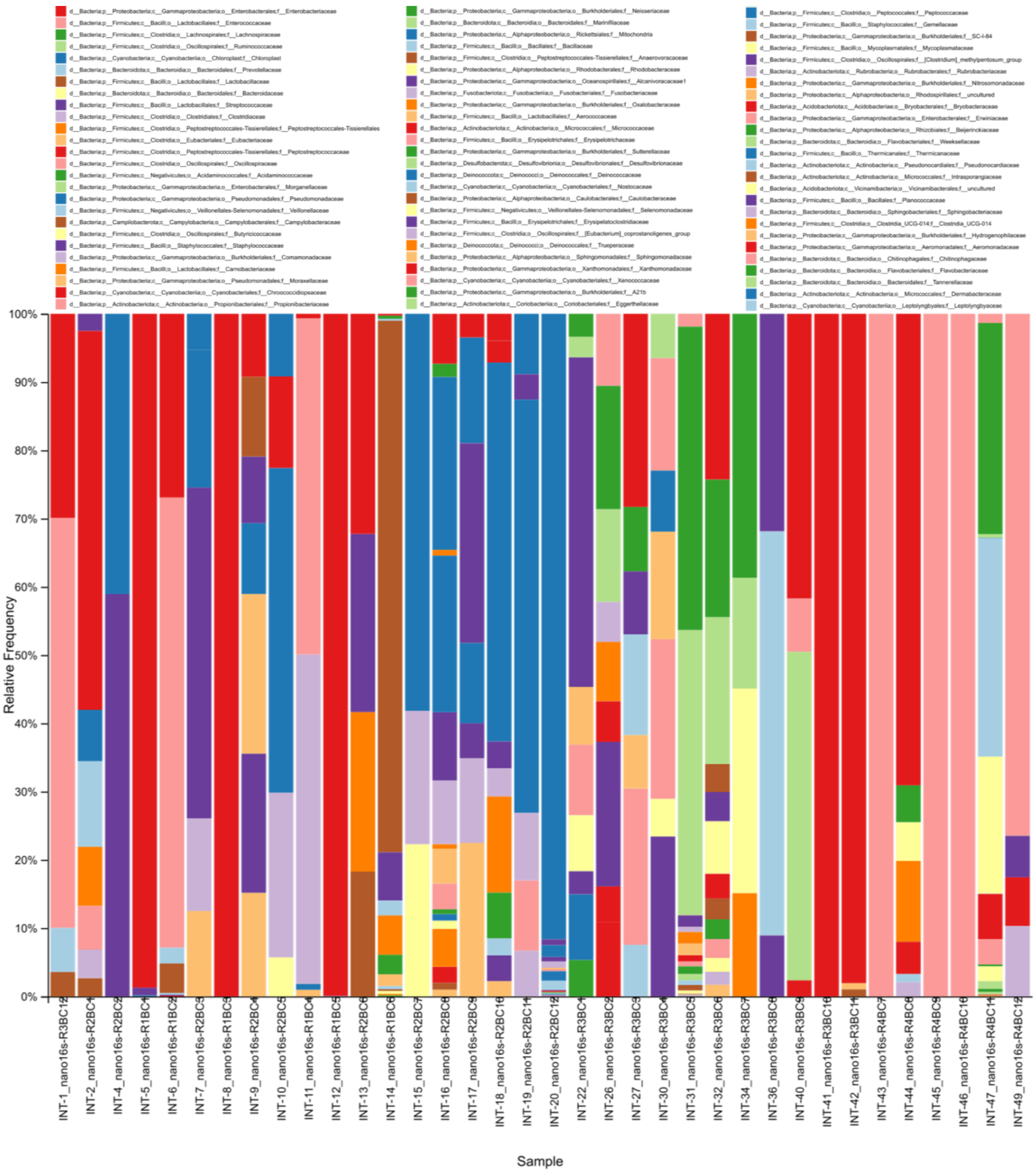

Supplementary Figure S7. Taxonomic composition of long-read sequencing data at the Family level

Bar plot showing the relative proportions of the families within all the samples based on 16S rDNA Nanopore sequencing. Initial reads demultiplexing and analysis was done on Nanopore Minknow, and taxonomic classification was carried out using the Bugseq pipeline.

Supplementary Table S1. Primers used for short-read and long-read amplicons generation

Sequencing platform	16s Region	Forward primer 5′-------- 3′	Reverse primer 5′-------- 3′	Reference
Nanopore (long read)	27F - 1492R	AGAGTTTGATCMTGGCTCAG	CGGTTACCTTGTTACGACTT	PMID: 33628638
Illumina (short read)	27F – 338R V1-V2	AGAGTTTGATCCTGGCTCAG	GCTGCCTCCCGTAGGAGT	PMID: 22123943
